# Nanoscale ordering of planar octupolar molecules for nonlinear optics at higher temperatures

**DOI:** 10.1038/s41598-021-81676-9

**Published:** 2021-01-26

**Authors:** Michał Jarema, Antoni C. Mituś, Joseph Zyss

**Affiliations:** 1 Department of Semiconductor Materials Engineering, Wrocław University of Science and Technology, Wybrzeże Wyspiańskiego 27, 50–370 Wrocław, Poland; 2 Department of Theoretical Physics, Wrocław University of Science and Technology, Wybrzeże Wyspiańskiego 27, 50–370 Wrocław, Poland; 3 LUMIN Laboratory and Institut d’Alembert, Ecole Normale Supérieure Paris-Saclay, CNRS, Université Paris-Saclay, 4, avenue des Sciences, Gif-sur-Yvette, France

**Keywords:** Nonlinear optics, Statistical physics, thermodynamics and nonlinear dynamics, Nanoscale devices

## Abstract

We develop scenarios for orientational ordering of an in-plane system of small flat octupolar molecules at the low-concentration limit, aiming towards nonlinear-optical (NLO) applications at room temperatures. The octupoles interact with external electric poling fields and intermolecular interactions are neglected. Simple statistical-mechanics models are used to analyze the orientational order in the *very weak poling* limit, sufficient for retrieving the NLO signals owing to the high sensitivity of NLO detectors and measurement chains. Two scenarios are discussed. Firstly, the octupolar poling field is imparted by a system of point charges; the setup is subject to cell-related constraints imposed by mechanical strength and dielectric breakdown limit. The very weak octupolar order of benchmarking TATB molecules is shown to emerge at Helium temperatures. The second scenario addresses the *dipoling* of octupolar molecules with a small admixture of electric dipolar component. It requires a strong field regime to become effective at Nitrogen temperature range. An estimation of the nonlinear susceptibility coefficient matrix for both scenarios is done in the high-temperature (weak interaction) limit formalism. We argue that moderate modifications of the system like, *e.g.*, an increase of the size of the octupole, accompanied by dipole-assisted octupoling, can increase the poling temperature above Nitrogen temperatures.

## Introduction

Organic molecules and materials have been of persistent interest throughout decades towards the exploration of nonlinear optical (NLO) phenomena and their progress towards applications^1–5^. The inherent tensorial properties at all scales promote symmetry considerations at the core of molecular nonlinear optics alongside propagative and quantum issues. Advances in nanoscale science and technologies have come to enable nonlinear optical configurations all the way from the wavelength scale of waveguided optics and microresonators^6^, down to the nanoscale^7–9^ and single molecule experiments^10^. Quadratic NLO processes require centrosymmetry breaking at the scales from individual molecules to bulk interactions in molecular crystals^11,12^. Polar conjugated molecules provide a versatile template, moreover embedded in the broader pool of multipolar non-centrosymmetric systems whereby octupolar molecules^13^ are a special case of major interest. Multipolar molecules and materials feature richer tensor potential towards more advantageous nonlinear polarization schemes, such as octupolar light–matter configurations abiding to polarization independence conditions^14,15^. Due to the symmetry induced net cancellation of their dipole moment that forbids classical dipolar coupling schemes, octupolar molecules have set a challenge since the early stage of their development. Therefore, the search and demonstration of efficient acentric orienting schemes for octupolar molecules remains an active domain of research to this day where theoretical modelling are spurring experiments and vice versa in a currently widely open context.

The first steps towards the evaluation of the required conditions for ordering of low-concentration octupolar molecules by electric field at nano-scale (nano-octupoling) were reported in Ref.^16^. A lattice system of planar two dimensional (2D) molecules with a single in plane rotational degree of freedom was investigated under the assumption of negligible molecular drift motion (i.e. fixed molecules restrained to rotate within their plane around their center of mass). In this study, the electric poling field was imparted by a system of electrodes. The molecular interactions as well as the influence of the polymer matrix on the ordering dynamics were neglected. It was found that effective poling of small octupolar molecules (octupoles) demanded irrealistic conditions, in the sub-Helium milikelvin temperature range. Such limitation was shown to result from two effects, namely the too small value of a geometric parameter (typical ratio of the sizes of the molecule and poling cell) as well as the spatially inhomogeneous orientation in the lowest energy state of the system. The latter issue was addressed^17,18^ by proposing an optimal symmetry adapted poling potential configuration of pure octupolar symmetry that matches the symmetry of the molecular species to be poled. However, this approach failed to lead to a significant increase of the poling temperature. Nevertheless, the aforementioned studies were just a starting point that relied on a heuristic model with *a priori* estimates of the relevant geometric and physical poling parameters.

**Figure 1:**
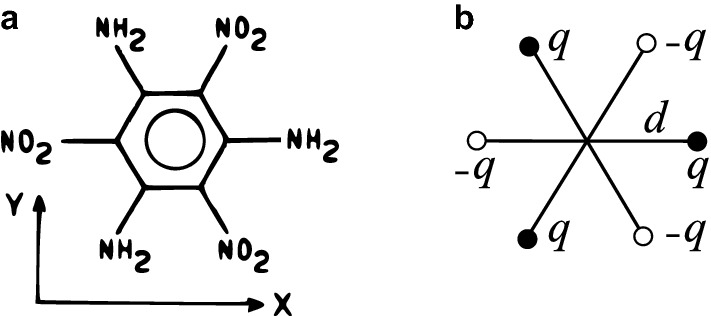
(**a**) An exemplary small octupolar molecule: TATB^19^ and (**b**) the model six-arm octupole.

The objective of this paper is to critically revise the requirements for effective ordering of octupolar molecules in the context of their NLO applications at higher temperatures. This includes (i) statistical-mechanics modeling of poling setups, (ii) estimation of available values of various physical parameters and (iii), evaluation of the amplitudes of NLO signals.

The paper is organized as follows. A classical model for an octupole is proposed and discussed. The poling setup and the corresponding electrostatic coupling energy are introduced. Statistical-physics aspects of octupoling (order parameters and the concept of very weak octupoling) are introduced and discussed. Octupoling conditions are extensively analyzed. Another scenario – dipoling of octupolar molecules – is discussed. Finally, the resulting magnitude of NLO signals is studied. A Quantum Chemistry (QChem) based validation of the classical model of octupoling proposed in this study is presented in the Supplementary Information.

## Octupolar molecule template

We model the molecular charge density distribution by point-charge extended multipoles of finite size constructed from the minimal number of point charges that is required to account for the given multipolar symmetry. The use of idealized point multipoles of infinitesimal size might be an over-simplification^20^ for separation distances comparable to the size of a molecule. There are two main model 2D octupoles^14^ with three-fold axial symmetry: a three-arm octupole considered previously^16^ and a six-arm octupole (6AO) shown in Fig. 1. The latter consists of six alternating charges $\pm q$ at ends of six arms of length *d*. The angle $\varphi$ between the *x* axis and a positively charged arm specifies the orientation of octupoles in 2D space. We focus on 6AO molecular template in view of its suitability to accommodate intermolecular interactions^21^. We point out that the model molecules are treated as rigid and non-polarizable.

The dipole and quadrupole moments of 6AO vanish, while the Cartesian components of its octupole moment are defined by

1 $$\begin{aligned} \mathcal {O}_{ijk}= \sum _{n=1}^6 q_n \, (\vec {r}_n)_i \, (\vec {r}_n)_j \, (\vec {r}_n)_k, \end{aligned}$$

where $q_n$ and $\vec {r}_n$ denote the charge and position of the *n*-th point charge in 6AO molecule. The octupole moment is a symmetric Cartesian tensor and is irreducible^22^. At the $\varphi =0$ orientation most of Cartesian components vanish, except for $\mathcal {O}_{xxx}=-\mathcal {O}_{xyy}=-\mathcal {O}_{yxy}=-\mathcal {O}_{yyx}=\tfrac{3}{2}qd^3$. Unlike the point octupole, the extended octupole template sustains also multipole moments of order higher than octupolar.

The norm of the octupole moment tensor expressed in Cartesian basis takes the following expression

2 $$\begin{aligned} \Vert \mathcal {O}\Vert =\sqrt{ \sum _{i,j,k} \left( \mathcal {O}_{ijk}\right) ^2 } =3qd^3. \end{aligned}$$

Based on the calculation of the octupole moment for a representative octupolar molecule TATB (see Supplementary Information) we use the following values of molecule’s parameters: size $d=2.44$ Å and molecular partial charge $q=0.66\,e$, where *e* is the elementary charge.

## Electric field induced nano-octupolar order

### Model of point charges octupoling

#### Poling setup

**Figure 2:**
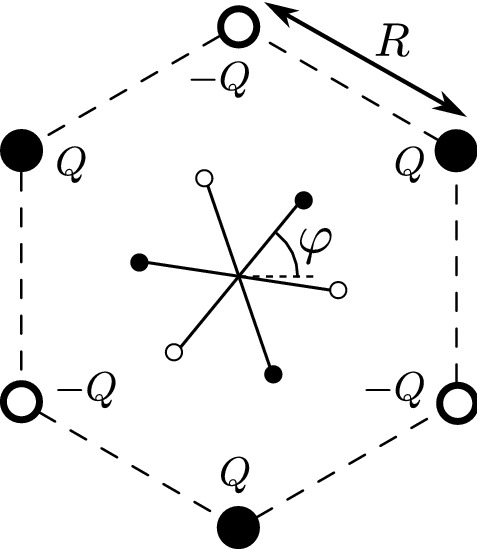
Octupoling setup: the model octupole with orientation angle $\varphi$ at the center of poling cell.

The octupolar field distribution is generated by a surrounding set of point charges. Figure 2 shows the octupoling cell consisting of six point charges of alternating signs $\pm Q$, which are located at the vertices of a regular hexagon of side *R*. The values of *R*, *Q*, limited by nano-scale fabrication technology and breakdown mechanisms, will be discussed in the following. This poling scheme differs from the classical electrode poling cell^16^.

#### Energy

The potential energy $E(\vec {r},\varphi )$ of the 6AO model molecule rotated by the angle $\varphi$ and subsequently translated from the cell center by a $\vec {r}$ vector is a sum of $6\times 6$ Coulomb interactions between molecular and poling charges. In particular, the energy $E(\vec {r}=0,\varphi )$ of a molecule located at the cell’s center is given by

3 $$\begin{aligned} E(\varphi )&= 6\sum _{m=0}^{5} \dfrac{(-1)^{m}\, k_0 \, q \, Q }{\sqrt{d^2+R^2-2dR\cos \left( \varphi - (\frac{1}{2}+m)\frac{\pi }{3} \right) }}, \end{aligned}$$

where the denominator is the distance between a selected poling charge and *m*-th molecular charge, and $k_0$ is the Coulomb constant. Taylor expansion with respect to the small parameter $d/R \approx 10^{-2}$ reads:

4 $$\begin{aligned} E(\varphi )&=\frac{\Delta \! E}{2} \sin 3\varphi + O\big ((d/R)^{9}\big ), \end{aligned}$$

where

5 $$\begin{aligned} \Delta \! E\simeq 45\,\frac{ k_0\,q\, Q }{R}\left( \frac{d}{R}\right) ^3 = 15 \,\frac{ k_0\, Q }{R^4} \,\Vert \mathcal {O}\Vert \end{aligned}$$

denotes the maximum–minimum energy difference and plays the role of energy barrier for a molecule at the cell center, oscillating around its ground state orientation $\varphi _0=\pi /2$. Thus, in the first approximation $E(\varphi ) \propto \sin 3\varphi$, as for the case of purely octupolar potential^17^. We conclude that the energy barrier $\Delta \! E$ at the center of the poling cell is proportional to the octupole moment of 6AO, and to the cell-related factor ${Q}/{R^4}$ that characterizes the amplitude of the octupoling field.

To test the reliability of a purely classical description of the molecule and its interaction with the poling electric field, we have calculated the energy of TATB molecule at the center of the poling cell using quantum chemistry simulations (Gaussian98, B3LYP/cc–pVDZ). We have found that the energy barrier agrees well (within 2%) with our classical model (see Supplementary Information for more details).

A brief description of the ground state (GS) is presented in Supplementary Information. The orientation of octupoles in GS is very similar to that in the electrode poling cell setup^16^. Consequences of inhomogeneous GS for octupoling have been discussed in Ref.^16^.

### Octupoling conditions: statistical mechanics analysis

#### Order parameter and its temperature dependence

The local orientational order parameter for a two-dimensional molecule with *n*-fold symmetry axis perpendicular to the plane where molecules are constrained to rotate can be conveniently defined as

6 $$\begin{aligned} p_n(\vec {r_k})= e^{i n \varphi _k}, \end{aligned}$$

where $\vec {r_k}$, $\varphi _k$ denote respectively the center of a *k*-labeled molecule and its orientation. For example, $n=1$ and $n=6$ correspond to a dipole and 2D hexagon^23^, respectively. A flat 6AO octupole has the three-fold symmetry ($n=3$); thus the corresponding order parameter reads^16^:

7 $$\begin{aligned} p_3(\vec {r_k}) = e^{3i\varphi _k}. \end{aligned}$$

The canonical average ${P_n}(\vec {r})$ of $p_n(\vec {r})$ is referred to as the average local order parameter:

8 $$\begin{aligned} {P_n}(\vec {r}) = \left\langle p_n(\vec {r}) \right\rangle = \frac{1}{Z}\,\int _0^{2 \pi /n} p_n(\vec r) e^{-\beta E(\vec r, \varphi )} {\,\mathrm d} \varphi , \end{aligned}$$

where $\beta =1/k_{\mathrm{B}}T$ (*T* is an absolute temperature) and *Z* denotes the partition function:

9 $$\begin{aligned} Z = \int _0^{2 \pi /n} e^{-\beta E(\vec r, \varphi )} {\,\mathrm d} \varphi . \end{aligned}$$

We will skip thereafter (unless otherwise stated) the index 3 for octupolar order parameters, thus $p = p_3$ and $P = P_3$. The overall order for *N* octupoles in the system is described by the average global order parameter

10 $$\begin{aligned} {{\mathbb {P}}} = \frac{1}{N}\sum _{i=1}^{N} P(\vec {r}_i). \end{aligned}$$

Let us estimate the degree of average local octupolar order at the center of the cell, see Fig. 2. We define the dimensionless inverse temperature

11 $$\begin{aligned} \beta ^\star = \frac{\Delta \! E}{2}\frac{1}{ k_{\mathrm{B}}\, T} = \frac{15\, k_0\, Q \, \Vert \mathcal {O}\Vert }{2\, R^4\,k_B\,T}, \end{aligned}$$

and use the first term in the expansion of the energy, Eq. (4). Then, the partition function is

12 $$\begin{aligned} Z = \int _{0}^{2\pi /3}\! e^{- \beta ^\star \sin 3\varphi } {\,\mathrm d}\varphi = \frac{2\pi }{3} I_0(\beta ^\star ), \end{aligned}$$

where $I_n$ stands for the modified Bessel function of the first kind^24^ for $n=0,1,2,...$. The average local order parameter reads

13 $$\begin{aligned} \begin{aligned} P&= \langle p \rangle = \langle e^{3 i \varphi } \rangle = \langle \cos 3\varphi \rangle + i\langle \sin 3\varphi \rangle \\&= 0 + i \frac{1}{Z} \int _{0}^{2\pi /3} \!\!\!\!\!\!\! \sin 3\varphi \, e^{- \beta ^\star \sin 3\varphi } \, {\,\mathrm d}\varphi = I_{1,0}(\beta ^\star )\,e^{3i\pi / 2}, \end{aligned} \end{aligned}$$

where $I_{1,0}=I_1/I_0$. The fluctuations of the complex local order parameter $p(\vec r)$, represented by variances of its real ($\mathfrak {R}\,p$) and imaginary ($\mathfrak {I}\, p$) parts, read

14 $$\begin{aligned} \begin{aligned} \mathrm{Var}\, \{\mathfrak {R}\,p\}&= \langle (\mathfrak {R}\,p)^2 \rangle - \langle \mathfrak {R}\,p\rangle ^2 = I_{1,0}(\beta ^\star ) / \beta ^\star , \\ \mathrm{Var}\, \{\mathfrak {I}\, p\}&=\langle (\mathfrak {I}\,p)^2 \rangle - \langle \mathfrak {I}\,p\rangle ^2 = 1 - I_{1,0}(\beta ^\star )^2 - I_{1,0}(\beta ^\star ) / \beta ^\star . \end{aligned} \end{aligned}$$

**Figure 3:**
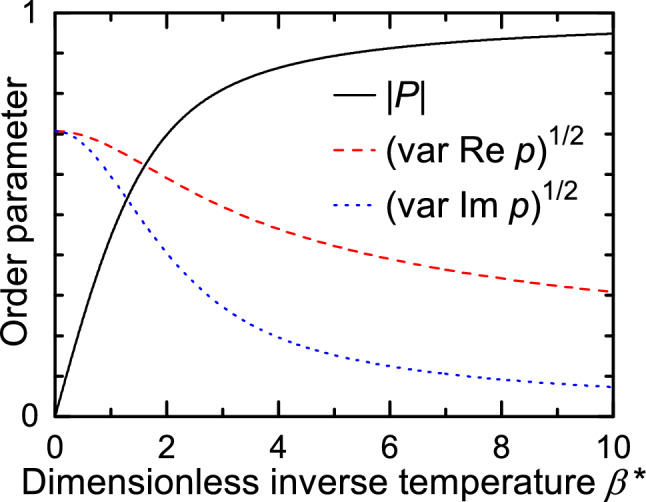
Characterization of local order *p* as function of dimensionless inverse temperature $\beta ^\star$: |*P*| (black line), $\sqrt{\mathrm{Var}\, \{\mathfrak {R}\,p\}}$ (red dashed line) and $\sqrt{\mathrm{Var}\, \{\mathfrak {I}\,p\}}$ (blue dotted line).

Figure 3 shows the plots of $|P|, \sqrt{\mathrm{Var}\, \{\mathfrak {R}\,p\}}, \sqrt{\mathrm{Var}\, \{\mathfrak {I}\,p\}}$ as a function of $\beta ^\star$. For $\beta ^\star \rightarrow \infty$ (low temperature) $P \rightarrow -i$, *i.e.* $|P| \rightarrow 1$. At low temperatures the amplitude |*P*| is close to 1, but the phase still fluctuates.

#### Very weak poling regime: energy barriers and experimental conditions

Consider a system with a rotational degree of freedom in an external poling field characterized by a potential well with energy barrier $\Delta E$. As long as thermal fluctuations are much lower than the energy barrier, *i.e.* $k_{\mathrm{B}}T \ll \Delta \! E$, the system is orientationally ordered. For $k_{\mathrm{B}}T \approx \Delta \! E$ the system is still moderately ordered but strong fluctuations come in. Finally, in the case when $k_{\mathrm{B}}T \gg \Delta \! E$ the fluctuations become completely disordering. Quite surprisingly, some NLO experimental effects, that directly relate to an average orientational order, can still be observed when the non-centrosymmetric order is negligible because of large fluctuations, thanks to the sensitivity and high signal-to-noise ratio of current detectors such as photomultipliers or photodiodes. Therefore, in parallel with the strong poling regime ($k_{\mathrm{B}}T \ll \Delta \! E$) and weak poling regime ($k_{\mathrm{B}}T \approx \Delta \! E$) we introduce the *very weak poling regime* ($k_{\mathrm{B}}T \gg \Delta \! E$), in which residual ordering can still be detected in current NLO experiments. In what follows we estimate the order of magnitude of corresponding (minimal) acentric order in the case of one of the most important NLO poling experiments that is the electric field induced second harmonic generation (EFISH)^25,26^.

The typical experimental conditions of EFISH in the case of simple dipolar push–pull molecules are as follows. The homogeneous poling electric field strength is of the order of dielectric strength of air $\mathcal {E}_\text {air}\approx 3\cdot 10^6\;\frac{\mathrm{V}}{\mathrm{m}}$. The ground-state dipole moment of dipolar push–pull molecules used in NLO is of the order of 10 D. For example, for pNA molecules with a dipole moment $\mu =6$ D^27,28^ the energy barrier reads

15 $$\begin{aligned} \Delta \! E_\text {EFISH}&= 2\, \mu \,\mathcal {E}_\text {air} \approx 0.75 \text { meV}, \end{aligned}$$

which compared to the the amplitude of thermal fluctuations $k_B T$ at room temperature ($T=300$ K) yields a factor $\alpha$

16 $$\begin{aligned} \alpha = \frac{k_{\mathrm{B}}\,300\,\mathrm{K}}{\Delta \! E_\text {EFISH}}&\approx {30}. \end{aligned}$$

The barrier is much lower than the amplitude of thermal fluctuations, therefore the acentric order in EFISH is very low from a statistical-mechanics point of view. Namely, in the two-state model the degree |*m*| of the orientational order is

17 $$\begin{aligned} |m| = \tanh \frac{\mu \mathcal {E}_\text {air}}{k_B T} = \tanh \frac{1}{2 \alpha } \approx 1.6\cdot 10^{-2}, \end{aligned}$$

which appears to be is sufficient for SHG detection.

Since the fundamental requirement of centro-symmetry breaking applies to both EFISH and octupoling, we expect that SHG due to the acentric order in a system of octupoles can occur at a similarly low magnitude of the order parameter^29^. Therefore, we propose a revised criterion for octupoling temperature in the very weak poling regime

18 $$\begin{aligned} T_\text {v.w.} = \alpha \frac{\Delta \! E}{k_{\mathrm{B}}}, \end{aligned}$$

instead of the weak criterion $T_{\mathrm{weak}} \approx {\Delta \! E}/{k_{\mathrm{B}}}$ from Ref.^16^. The value of the octupolar order parameter in the very weak poling regime is obtained by evaluating Eq. (13) at $(\beta ^\star )_\text {v.w.}=\frac{1}{2\alpha }$ (compare Eqs. (11) and (18)):

19 $$\begin{aligned} |P|_\text {v.w.} \approx 10^{-2}, \end{aligned}$$

in fair agreement with its dipolar counterpart, Eq. (17).

**Figure 4:**
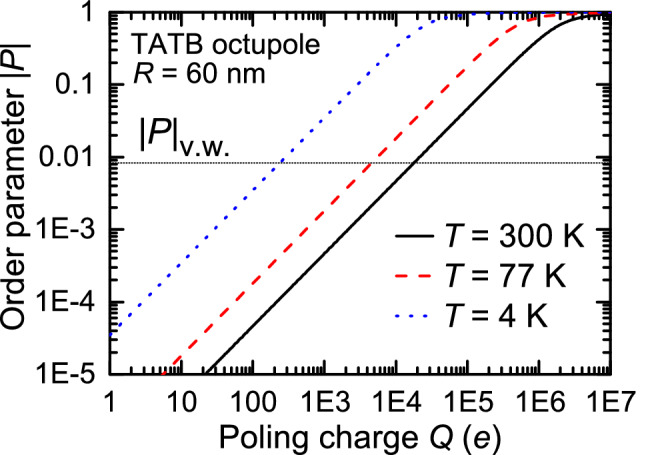
Order parameter |*P*| in the central part of a point charge poling cell in function of the poling charge *Q* for selected temperatures. The order parameter $|P|_\text {v.w.}$ for the very weak regime is represented by the horizontal line.

#### Discussion of octupoling conditions

Based on previous considerations, we discuss now the conditions for effective octupoling in the central region of a realistic poling cell, in the very weak poling regime, Eq. (19). In order to increase the poling temperature the cell-related factor $Q/R^4$, see Eq. (5), has to be maximized; therefore we look for small values of *R* and large values of *Q*. In what follows we use the same poling cell size $R=60$ nm as in Refs.^16,30^, and concentrate on the analysis of physical constraints imposed by the magnitude of the poling charge *Q*.

Figure 4 shows the double logarithmic plot of order parameter $|P|=I_{1,0}(\beta ^\star (Q,T))$ (Eq. (13)) vs. *Q* for a few selected temperatures - liquid Helium, liquid Nitrogen and room temperature. It implies the power law dependence for |*P*|:

20 $$\begin{aligned} |P|(Q, T) = f(T)\,Q^{x}. \end{aligned}$$

The exponent *x* and function *f* can be easily inferred in the high temperature expansion (HTE) limit when $\beta ^\star \rightarrow 0$. Namely, in this limit $I_{1,0}(\beta ^\star ) \approx \frac{1}{2} \beta ^\star \propto \frac{Q}{T}$. Hence, $x=1$ and $f(T) \propto 1/T$. A closer inspection of the plot shows that those results hold for, say, $|P| \le 0.5$, far beyond the very weak order regime. Putting $|P| = |P|_\text {v.w.}$ in this formula and using the definition of $\beta ^{\star }$ in Eq. (11) provides the charge $Q_\text {v.w.}$ which grants the very weak poling conditions at temperature *T*:

21 $$\begin{aligned} Q_\text {v.w.} = \frac{4}{15}\,\frac{k_B R^4}{k_0 \Vert \mathcal {O}\Vert }\, |P|_\text {v.w.}\,T. \end{aligned}$$

The practical implementation of very weak octupolar poling conditions at temperature *T* is limited by the restrictions imposed on the cell setup by the acceptable magnitude of the accompanying electric field $\mathcal{E}(Q_\text {v.w.})$. Two physical effects appear to be of primary importance.

Firstly, the dielectric strength of the medium must prevent electric breakdown. In practice, the “point” poling charges are small charged spheres of radius $r_s$. The electric field $\mathcal {E}_s$ close to the surface of a sphere bearing charge *Q* is

22 $$\begin{aligned} \mathcal {E}_s(Q)=\frac{k_0 Q}{{r_s}^2}. \end{aligned}$$

The dielectric strength of the matrix has to be higher than $\mathcal {E}_s(Q)$, which sets an upper boundary $Q_{s,max}$ on *Q*: $Q < Q_{s,max}$.

Secondly, Coulomb forces between poling charges must be balanced by elastic reaction forces, so as to prevent collapse of the poling cell. The net Coulomb force acting on any of the poling charges due to the remaining five ones (see Fig. 2) is directed towards the center of the poling cell and is given by

23 $$\begin{aligned} F(Q) = c_F \frac{k_0 Q^2}{R^2} , \; c_F=\frac{15-4\sqrt{3}}{12} \approx 0.67. \end{aligned}$$

The charged spheres of radius $r_s$ interact through a surface $S\approx \pi {r_s}^2$ with the surrounding medium. The resulting pressure is:

24 $$\begin{aligned} \eta (Q)=\frac{F}{S}=c_F \frac{k_0 Q^2}{\pi {r_s}^2 R^2}. \end{aligned}$$

To avoid the mechanical breakdown of the polymer matrix its tensile strength has to be larger than $\eta (Q)$, which sets another upper limit $Q_{\eta , max}$ on the magnitude of *Q*: $Q < Q_{\eta , max}$.

**Figure 5:**
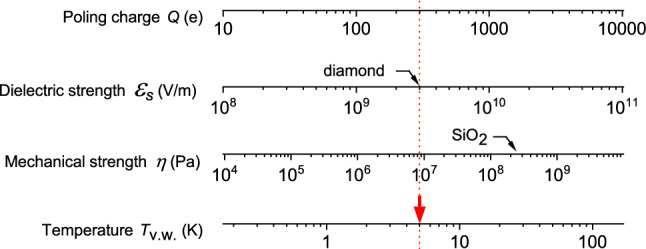
Mutual relations between the poling charge *Q*, the dielectric strength $\mathcal {E}_s(Q)$ (Eq. (22)) and mechanical strength $\eta (Q)$ (Eq. (24)), and the temperature *T*(*Q*) (Eq. (21)) for octupoling at the cell’s center in the very weak poling regime for $R=60$ nm and $r_s=12$ nm. Black arrows design maximal accessible values (dielectric and mechanical strengths) of the poling setup. The red arrow points at maximal accessible temperature $T_\text {v.w.}$ resulting from the restrictions imposed by $\mathcal {E}_s$.

The mutual relations between the poling charge *Q*, the electric field amplitude $\mathcal {E}_s(Q)$ (Eq. (22)), the pressure $\eta (Q)$ (Eq. (24)) and temperature *T*(*Q*) (Eq. (21)) are summarized in Fig. 5. They are useful for an evaluation of very weak poling conditions at given temperature *T*. To this end, the parameters $Q_{s,max}$ and $Q_{\eta ,max}$ have to be estimated.

Let us discuss $Q_{s,max}$ first. The dielectric strength of common polymers is at least one order of magnitude higher than the dielectric strength of air^31,32^. Moreover, it usually increases for small thicknesses of the insulating layer, short timescale, low temperature and for high purity materials^33^. Interestingly, thin films (170 nm) of thermally-cured DNA–CTMA sol–gel have been demonstrated^34^ to sustain an electric field as high as $9\cdot 10^8\frac{\mathrm{V}}{\mathrm{m}}$ at room temperature. Moreover, the spheres can be covered with an insulating layer to increase the threshold for electric breakdown. An outstanding value of dielectric strength is the avalanche breakdown strength of chemical vapor deposited diamond^35–38^. It has been reported^38^ that an electric field up to $5 \cdot 10^9~\frac{\mathrm{V}}{\mathrm{m}}$ can be applied to a 200 nm-thick diamond. To estimate the threshold parameter $Q_\text {s,max}$ we set $\mathcal {E}_s$ to a slightly lower limit of $3 \cdot 10^9~\frac{\mathrm{V}}{\mathrm{m}}$ (at the onset of breakdown^38^) and use the value $r_s=R/5$ for the radius of the sphere. We find, from Eq. (22), $Q_{s,max} \approx 300\,e$.

Next, let us estimate the mechanical breakdown effect related to pressure. Tensile strength of PMMA is about 50-75 MPa at room temperature^39^. The corresponding upper limits for the poling charge $Q_{\eta ,max}$ are correspondingly in the 700 *e* to 900 *e* range. Moreover, the charged spheres can be mechanically supported by a skeleton made from a harder material, *e.g. *fused silica ($\text {SiO}_2$) with tensile strength of about 150 MPa^40^. Then, $Q_{\eta ,max}$ can raise up to $1250\,e$.

To summarize, the critical restrictions on *Q* result from the electric breakdown effect:

25 $$\begin{aligned} Q< Q_{s,max} \approx 300\,\mathrm {e} < Q_{\eta ,max}. \end{aligned}$$

A gold sphere of radius $r_s= R/5=12$ nm contains about $4\cdot 10^5$ atoms and $4\cdot 10^3$ laying on its surface, so charging it with 300 electrons results in a realistic charge density.

Finally, inserting $Q_\text {v.w.} = Q_{s,max} = 300$ e and $|P|_\text {v.w.} = 10^{-2}$ into Eq. (21) we find that the very weak octupoling can take place in the liquid Helium range:

26 $$\begin{aligned} T_\text {v.w.}\approx 5~K. \end{aligned}$$

### Dipoling-induced octupolar order

A different way of promoting octupolar orientational order is to consider molecules of mixed dipolar–octupolar character, to be poled by a strong dipolar electric field.

By way of introducing a quantitative model, let us start with a qualitative presentation. It is well known that symmetry arguments strictly forbid octupoling of dipolar–octupolar molecules via a homogeneous external field in the weak poling regime, *i.e.*, when the Boltzmann factor is expanded to the first order w.r.t. the inverse temperature $\beta$^15,30^. However, higher order expansion terms may exhibit the symmetry features of octupolar order parameter and therefore promote a non-zero octupolar order. For a mixed dipolar–octupolar molecule sustaining a strong octupolar second polarizability tensor component, SHG anisotropy may exhibit properties with octupolar symmetry features. A more technical development of this situation is given in the next Section.

More generally, we wish to point out that such a mechanism complies with the basic distinction between low- and high-field effects that pervades throughout nonlinear optical phenomenology. In a similar context it has been shown both theoretically and experimentally that the template mixed dipolar–octupolar molecule 1,3-dinitro-4,6-di-(n-butylamino)-benzene (DNDAB, with $C_{2v}$ point-group symmetry, see Ref.^15^) exhibits a significant departure of its nonlinear anisotropy from the weak poling field value of 3, onto lower values that are indicative of strong octupolar contributions to nonlinear susceptibility^41,42^. In addition to the usual dipolar component, SHG signal has been shown to sustain octupolar properties.

Let us formulate this scenario in a quantitative way. Consider a planar dipolar–octupolar molecule abiding to an in-plane two-fold symmetry axis and a dipole moment along that axis (*i.e. *in $C_{2v}$ symmetry, lowering the symmetry of an equilateral triangle into that of an isosceles one), in an external homogeneous field $\vec {\mathcal {E}}$. Generalization to the case where the poling field exhibit an octupolar component is briefly considered in the Discussion. The energy $E(\varphi )$ of an octupolar molecule with attached dipole moment $\vec {\mu }$ in this case originates exclusively from the dipole–field interaction, since the field $\vec {\mathcal {E}}$ is not allowed to couple, due to symmetry arguments, to the octupolar electric moment of the molecule^15,30^. $E(\varphi )$ depends on the orientation $\varphi$ of the dipole (note different energy formula from that in Eq. (13)):

27 $$\begin{aligned} E(\varphi )=-\vec {\mathcal {E}}\cdot \vec {\mu }=-\mathcal {E}\mu \cos \varphi . \end{aligned}$$

**Figure 6:**
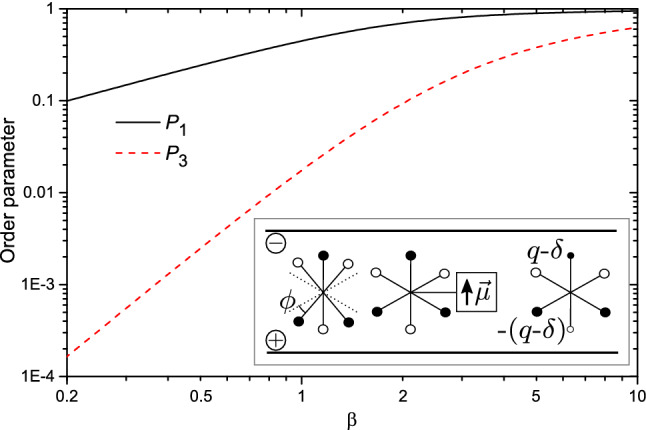
Log-log plot of octupolar order parameters $P_1$ (black solid line) and $P_3$ (red dashed line) in function of the inverse temperature $\beta ^*_d$. Inset: modified dipolar–octupolar molecules, see text.

The average local order planar parameters $P_n$, Eq. (8), read:

28 $$\begin{aligned} \begin{aligned} P_n&= \langle e^{i n \varphi }\rangle = \frac{1}{Z} \int _0^{2 \pi } e^{i n \varphi } e^{- \beta \,E(\varphi )} {\,\mathrm d}\varphi \\&= \frac{1}{Z}\int _0^{2 \pi } \cos (n \varphi ) e^{ \beta ^\star _d \cos \varphi } {\,\mathrm d}\varphi = \frac{I_n(\beta ^\star _d)}{I_0(\beta ^\star _d)}, \end{aligned} \end{aligned}$$

where $Z = \int _0^{2 \pi } e^{ \beta ^\star _d \cos \varphi } {\,\mathrm d}\varphi$ and $\beta ^\star _d =\frac{\mathcal {E}\mu }{k_B T}$. The plots of dipolar and octupolar order parameters $P_1$ and $P_3$ in function of $\beta ^\star _d$ are shown in Fig. 6. At low temperatures $P_3 \approx (P_1)^9$ (low temperature expansion) and both parameters have comparable values when $P_1$ is close to 1. The situation becomes very different at high temperatures (weak interaction limit) – the leading terms in this expansion read

29 $$\begin{aligned} P_n\simeq \frac{(\beta ^\star _d)^n}{2^n n!}. \end{aligned}$$

**Figure 7:**
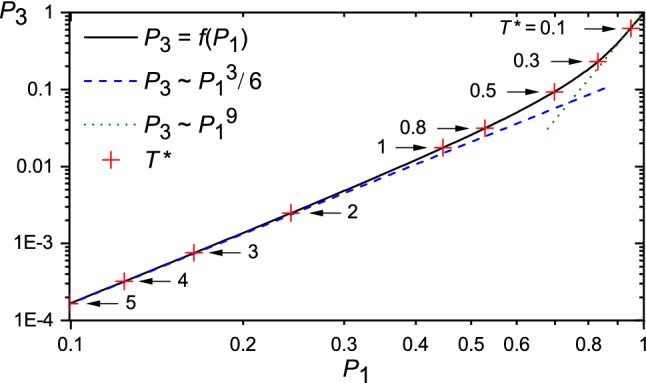
Log-log plot of parametric relation between order parameters $P_3$ and $P_1$ (black solid line). The reduced temperature parameter $T^\star =1/\beta ^\star _d$ is marked along the curve. The high-temperature asymptote $P_3=(P_1)^3/6$ (blue dashed line) and low-temperature asymptote $P_3=(P_1)^9$ (green dotted line) are also shown.

In particular, the octupolar order parameter $P_3$ vanishes in the first order of high-temperature expansion, in agreement with our former statement. However, it becomes non-zero for the third order expansion, while relating to the dipolar order parameter $P_1$ in the following way:

30 $$\begin{aligned} P_3\simeq (P_1)^3/6, \end{aligned}$$

indicating that the octupolar order parameter becomes negligibly small in comparison with the dipolar one when $\beta ^\star _d \ll 1$. On the other hand, a sufficiently strong homogeneous field (such that $P_1 \approx 1$) promotes a high degree of in-plane octupolar order. The relation between order parameters $P_3$ and $P_1$ for a range of temperatures is summarized in Fig. 7.

Let us estimate the magnitude of the dipolar moment necessary to support the very weak poling regime $(P_3)_{\text {v.w.}} = 0.01$, Eq. (19), at Nitrogen temperatures ($T=77$ K). Using Eq. (29) and the definition of $\beta ^\star _d$ we find

31 $$\begin{aligned} T \simeq \frac{\mathcal {E}\mu }{k_B \root 3 \of {48 (P_3)_{\text {v.w.}}}} \approx 1.28 \frac{\mathcal {E}\mu }{k_B} \approx 61.7\, \tfrac{\mathrm{K}}{\mathrm{D}}\, \mu , \end{aligned}$$

with $\mathcal {E}=2\cdot 10^8$ V/m as available poling field strength^31^; $\mu$ is expressed in Debye unit. Upon replacing *T* with 77 K we get $\mu \simeq 1.25$ D. The related dipolar order parameter $P_1$ is, contrary to $P_3$, not negligible: $P_1 \approx 0.36$. To create such a dipole the 6AO molecule has to be modified accordingly. Let us discuss briefly three strategies: (i) distorting the shape of the octupolar molecule, (ii) adding a peripheral dipolar group, and (iii) modifying the molecular charge density (see inset in Fig. 6).

In the first case four arms of a 6AO molecule are bent towards its $C_2$ axis (formed by remaining two arms) by $\phi =2.7^\circ$. Such a distortion removes the $C_3$ symmetry axis but leaves the $C_2$ unchanged (i.e. lowers symmetry from $D_{3h}$ to $C_{2v}$) and decreases the irreducible octupole moment by about 1% while inducing a dipole moment of 1.25 D.

In the second strategy, a group with a small dipole moment is rigidly attached to an octupolar molecule in such a manner that it is separated from the $\pi$-conjugated system responsible for octupolar hyperpolarizability (in a similar way to the CN nitrile group in the NPAN molecule^15^). For example, it could be accomplished by a substitution of one C–H bond by a more polarized C–Cl bond. To estimate the magnitude of the corresponding dipole moment, we have analyzed experimental dipole moment data^43^ for 18 simple compounds consisting of carbon, hydrogen, and a single chloride atom adjacent to a carbon atom, such that with H replacing Cl their dipole moment would vanish due to symmetry. The average and standard deviation for those compounds is $\mu _\text {CCl}=(1.95\pm 0.23)$ D. Similar query for 14 compounds containing a single CN group yields $\mu _\text {CN}=(3.96\pm 0.30)$ D.

In the third template the magnitude of two $\pm q$ opposite charges in the 6AO molecule is reduced by $\delta =0.08 \, q$. It leads again to $C_{2v}$ symmetry and decreases the irreducible octupole moment by 2.5% while inducing a dipole moment of 1.25 D. This method is inspired by the generalized equivalent internal potential model^44^. Namely, in the TATB molecule one pair of $\mathrm {NO}_2$ and $\mathrm {NH}_2$ donor and acceptor groups (which are strong substituents in nonlinear regime^44^) are exchanged with other two slightly less “pushing” and “pulling” substituents.

We point out that the modification of the structure of purely octupolar molecule is, in general, accompanied by a change of the octupolar component of the molecule’s second hyperpolarizability. On the other hand, taking into account the fact that the structural modifications as well as the changes of irreducible octupolar moments are minor, we assume that the octupolar non-linear responses are also modified to a low degree.

We conclude that the very weak octupolar order can be reached by dipoling of dipolar–octupolar molecules with $\mu =1.25$ D at liquid Nitrogen temperatures:

32 $$\begin{aligned} T_\text {v.w.} = 77 \text { K}. \end{aligned}$$

## SHG coefficients for different scenarios for promoting nano-octupolar order

Let us estimate the values of some experimentally-accessible NLO parameters in the previously discussed poling scenarios. To this end, we apply the methodology worked out in Ref. ^30^, aiming at the calculation of NLO susceptibilities of a system of multipolar molecules ordered by multipolar electric field in the weak interaction (HTE) limit. Namely, the average of any tensor property attached to a molecule (e.g. its charge distribution) can be expressed as a weighted sum of irreducible tensorial parts of the property, weighed by multipolar local order parameters $P_n = \langle p_n \rangle$ (Eq. (8)). For example, the quadratic nonlinear susceptibility tensor ${\tilde{\chi }}^{(2)}$, defined as the product of number density $\mathcal {N}$ and quadratic hyperpolarizability tensor ${\tilde{\beta }}$ averaged over molecular orientations, reads in 2D^30^

33 $$\begin{aligned} {\tilde{\chi }}^{(2)}(\vec {r})=\mathcal {N}\langle {\tilde{\beta }}\rangle =\mathcal {N}\, \sum _{n=0}^{3} P_n(\vec r) \,{\tilde{\beta }}^{J=n}, \end{aligned}$$

where tildes denote tensors. The sum runs over the irreducible parts *J* of the tensorial property ${\tilde{\beta }}$ – from $J=0$ (scalar part) to the rank of the tensorial property $J=3$ for ${\tilde{\chi }}^{(2)}$. Moreover, a symmetric tensor, like ${\tilde{\beta }}$ in Kleinmann regime, consists only of irreducible parts with *J* displaying the same parity as the rank of the tensor^22^. Thus, the NLO response consists of vectorial ($J=1$) and octupolar ($J=3$) parts:

34 $$\begin{aligned} {\tilde{\chi }}^{(2)}(\vec {r})=\mathcal {N}\,\big (P_1(\vec r) \,{\tilde{\beta }}^{J=1} + P_3(\vec r) \,{\tilde{\beta }}^{J=3}\big ). \end{aligned}$$

For a three-fold-symmetric octupolar molecule like TATB, the vectorial part ${\tilde{\beta }}^{J=1}$ vanishes. In the case of dipolar induced octupolar order the vectorial part becomes, in general, non-zero, and can dominate the response as $P_3 \ll P_1$ in the weak interaction limit, see Fig. 6. However, the octupolar part of the response offers advantages over its vectorial counterpart due to its richer tensorial structure^14,15^. In particular, while the octupolar response in a given (vectorial part) direction can be dominated by its dipolar counterpart, it remains uninfluenced in two other directions. In other words, the optical waves in different directions can still be nonlinearly coupled by non-zero off-diagonal tensor components like $\chi ^{(2)}_{xyy}, \chi ^{(2)}_{yxx}$. In what follows, we focus on octupolar order and proceed to estimate the value of

35 $$\begin{aligned} {\tilde{\chi }}^{(2)}(\vec {r})=\mathcal {N}\, P_3(\vec r) \,{\tilde{\beta }}^{J=3}. \end{aligned}$$

Using the overall global order parameter $\mathbb {P}$, Eq. (10), instead of $P_3$ in Eqs. (33)–(35) corresponds to averaging over the positions $\vec {r}$ in the cell, and yields the global susceptibility of the system ${\tilde{\chi }}^{(2)}$. A related, more widely used parameter, is the nonlinear susceptibility coefficient matrix^1^ $d_{il}=\tfrac{1}{2\varepsilon _0}\chi ^{(2)}_{ijk}$ ($\varepsilon _0$ is vacuum permittivity, Voigt’s index notation^1^ is used), for which we get

36 $$\begin{aligned} d_{il}=\frac{\mathcal {N}}{2\varepsilon _0}\, \mathbb {P} \, \beta ^{J=3}_{ijk}. \end{aligned}$$

Equations (33)–(36), which were derived within the weak interaction limit, can as well be applied for order parameters obtained by other means, provided that the weak interaction approximation remains valid – in particular, in the very weak poling limit used in this paper.

Let us estimate the order of magnitude of the $d_{il}$ tensor components for TATB guest molecules homogeneously dispersed in a PMMA polymer host matrix with weight fraction $f_{\mathrm{wt}}$. The number density is $\mathcal {N}=f_{\mathrm{wt}} \rho / m_1$, where $\rho$ denotes the density of the system and $m_1$ stands for the mass of one molecule. It is useful to estimate the corresponding average intermolecular distance *l* between guest molecules. For uniformly mixed system $l \approx \mathcal {N}^{-1/3}$, and the reduced distance $L = l/d$ reads

37 $$\begin{aligned} L=(f_{\mathrm{wt}} \rho / m_1)^{-1/3} d^{-1} \approx 2.92 / \root 3 \of {f_{\mathrm{wt}}}, \end{aligned}$$

with $m_1=258$ u (atomic mass unit), $d=2.44$ Å for a TATB molecule and $\rho =1.18~\text {g}/\text {cm}^3$ for pure PMMA polymer matrix. Next, the components of the $\beta ^{J=3}_{ijk}$ hyperpolarizability of a TATB molecule in the reference frame of Fig. 1 are estimated as^14,45^ $\beta _{xxx}=-\beta _{xyy}=10^{-29}\text { [esu]}\approx 3.5\cdot 10^{-50}\,\tfrac{(C\,m)^3}{J^2}\text { [SI]}$.

The results are summarized in Table 1, where we use three values of intermolecular distance $L = 5, 10$ and 15. The first one corresponds to $f_{\mathrm{wt}}\approx 0.2$ which is a high but achievable concentration in host–guest poled polymer systems^31^. A short comment on this choice is given in the Discussion. Two simplifications were made. Firstly, in the case of point charge octupoling the order is inhomogeneous and, for simplicity, only the central region of the cell is considered, where $\mathbb {P}\approx P(r=0)$. Secondly, in the case of dipoling-assisted scenario we have neglected the influence of added dipolar moment on the hyperpolarizability $\beta _{ijk}$, see comment at the end of the previous Section.

**Table 1:** NLO susceptibility tensor ${\tilde{d}}=(2\varepsilon _0)^{-1}{\tilde{\chi }}^{(2)}$ in the very weak poling regime, Eq. (36), for a few values of relative distance *L*, weight fraction $f_{wt}$ and concentration $\mathcal{N}$. The non-zero coefficients of $d_{il}$ are $d_{11}=-d_{21}=-d_{26}$.

L	$f_{wt}$	$\mathcal{N}$ [$\hbox {cm}^{-3}$]	$d_{11}$ [pm/V]
5	20%	$5 \cdot 10^{20}$	$1.5\cdot 10^{-2}$
10	2.5%	$6 \cdot 10^{19}$	$1.9\cdot 10^{-3}$
15	0.7%	$2 \cdot 10^{19}$	$5.6\cdot 10^{-4}$

For comparison, coefficients $d_{il}$ for NLO crystals are typically of the order of 0.5 pm/V (quartz) to 70 pm/V (GaSe). For dipoled host–guest system in polymer matrix the achievable values are 2.5 pm/V (DR1, 2.74 wt% in PMMA) to 84 pm/V (modified DR1, 10 wt% in PMMA)^31,46^. In the next Section we discuss briefly some scenarios which result in a substantial increase of the value of this parameter in our model.

## Summary and discussion

We have studied two scenarios of orientational ordering of small flat octupolar and slightly modified octupolar molecules by external electric field in the context of quadratic non-linear optics phenomena with emphasis on second harmonic generation. While we have used a simple statistical mechanics modeling, a new methodological approach was applied to the study of the very weak poling regime, when the emerging overall orientational order is much smaller than thermal fluctuations. Experiments should be enabled by the improved sensitivity and higher signal-to noise ratio of current detectors used in NLO experiments.

Our method of analysis of very low orientational octupolar order encompasses both statistical mechanics modeling and an estimate of material limitations resulting from current nanotechnologies, with detailed calculations performed for the TATB molecule. However, the method is very general and can be applied to larger molecules (see below), metal-organic complexes, nanocrystals, nanostructures etc.

We have found that the current nano-scale technology limits the octupoling temperatures for small octupolar molecules, like TATB, to a few Kelvins. This improves the previous estimations^16^ by four orders of magnitude. Its origin is as follows: (i) increase of the poling temperature by factor $\alpha \approx 30$ due to the very weak poling scheme, (ii) five times larger octupole moment and (iii) much stronger octupoling electric field. Namely, the electric field strength in the middle of the poling cell would correspond to the voltage $V_0 = \frac{15\pi }{16}\frac{ k_0\, Q }{R} \approx 35\text { V}$ in the electrode poling scenario^16^, where the value $V_0=0.1$ V has been previously used. The combination of those numbers yields around $5\cdot 10^4$ – a factor by which the poling temperature was underestimated in Ref.^16^.

In the case of dipoling – a scenario in which modified octupoles with a small dipolar moment are poled by a strong homogeneous electric field – the very weak octupolar order is preserved up to liquid Nitrogen temperatures. The characteristic feature of this scenario is a trade-off between an increase of the poling temperature, proportional to $\mu$, Eq. (31), and the loss of the purely octupolar component of the NLO response. The numerical estimations were based on the assumption that the emergence of a small dipolar moment does not influence the octupolar hyperpolarizability. This hypothesis is based on the model^44^ of equivalent internal potentials acting on the polarizable molecular skeleton from the surrounding push–pull substituent sites. Both permanent moments and corresponding irreducible parts of the hyperpolarizability are linear responses to the internal potentials, and are therefore expected to change in the same proportion for a small perturbation of the molecular structure. In particular, a correlation between calculated $(J=3, m=\pm 3)$ components of $\tilde{\mathcal {O}}$ and ${\tilde{\beta }}$ has been reported^44^. A quantitative analysis of this topic requires quantum chemistry calculations for the molecules of interest and goes beyond the scope of this paper. Another advantage of this scenario is a strong increase of octupolar order at lower temperatures. At Helium temperatures ($T=5$ K) the order parameters are $P_1 \approx 0.96, P_3 \approx 0.68$. The latter indicates a high degree of octupolar order, some two orders of magnitude larger than in the very weak poling limit. This yields, in the first approximation, an increase of SHG by an order of magnitude: $d_{11} \approx 0.1$ pm/V for $f_{wt} = 20\%$, see Table 1.

The results of this study imply that a moderate change of the parameters of the model can raise the octupoling temperature to a more acceptable range. We postpone a systematical analysis to future studies and will limit ourselves here to the discussion of some emerging scenarios. Consider first the pure octupoling method. Technological constraints impose an upper limit to the poling charge $Q_{s, max}$ (Eq. (25)). This introduces (see Eq. (21)) a relation between the poling temperature, the octupolar order parameter and the size of the poling cell in the very weak poling regime:

38 $$\begin{aligned} T = \text {const}\,\frac{\Vert \mathcal {O}\Vert }{R^4\, |P|_\text {v.w.}} \propto \frac{|q|\,d^3}{R^4\, |P|_\text {v.w.}}, \end{aligned}$$

since $\mathcal {O}\propto qd^3$. We find that the most promising method to increase the poling temperature is to increase the octupolar moment of the molecule. For example, a twofold increase of charge *q* accompanied by a twofold increase of *d* (thus the size of a molecule) brings the octupoling temperature to the Nitrogen range; if the size were to be increased by a factor of three, the poling temperature would still be below room temperature but close to it. The corresponding molecular design provides an interesting target for quantum chemistry calculations. Other than that, reduction of the detection limit of octupolar order *P* below the $P\approx 1\%$ threshold would also allow for an increase of the poling temperature.

Another interesting scenario not discussed in this paper is the dipole-assisted octupoling, i.e., in the presence of both dipolar and octupolar poling fields which jointly enhance the octupolar order. When both octupolar-order components (due to dipolar and octupolar fields) are in the very weak poling regimes, then the resulting octupolar order is the sum of both components. This is, e.g., the case for larger molecules discussed above, with octupoling temperature in Nitrogen region, the net order remaining in the very weak limit, leading to a very low NLO response. On the other hand, when both components are not small then the net octupolar order is larger than the sum of both components, due to nonlinear interaction term. Consider again the larger molecule discussed above (with octupole moment increased by factor 16 and dipole moment $\mu = 1.25$ D) now at Helium temperature ($T=5$ K). Then, the net octupolar order parameter $P_3$ has two components: the dipoling-induced octupolar order parameter $P_3 \approx 0.68$ and the octupoling-induced one $P_3 \approx 0.13$. The sum yields around 0.8, but the nonlinear effects will still increase it, approaching the limit of perfect octupolar order $P_3 = 1$. In this limit the nonlinear susceptibility tensor ${\tilde{\chi }}^{(2)}$ (Eq. (33) is directly proportional to the quadratic hyperpolarizability tensor ${\tilde{\beta }}$. For TATB molecules we find $d_{11} \approx 1$ pm/V for $f_{wt} = 20\%$. Detailed analysis of this topic goes beyond the scope of this paper.

The next step in the modeling of octupoling is to account for the three-dimensional geometry of more general molecular octupoles. The description of orientational order in three dimensions requires a more advanced mathematical formalism. This extension is currently under investigation and will be reported later.

In the current study the octupolar molecules are subject to interactions with electric poling field only, while intermolecular octupole–octupole interactions are neglected in the low-density limit. Preliminary results show that they become important for $f_{wt}$ larger than a few percent (*L* lower than, say, 10). The interactions introduce local correlations which modify the local and global order and can result in an increase of poling temperature. The discussion presented above shows that the modification of non-linear optical parameters of octupolar molecules also results in an increase of the poling temperature. While at present no quantitative estimations of those effects are available, we believe that nano-poling at room temperatures is achievable. This, in turn, would afford the orientational mobility of the guest molecules as a result of heating the matrix above its glass temperature, used in traditional poling procedure.

In this study the matrix is not a dynamical system – it serves only to estimate cell-related constraints which limit the feasibility of nano-poling not only in low, but also at higher poling temperatures in future applications. The choice of PMMA matrix was not specific – it was chosen as one of typical polymers used in nonlinear optics applications. Other than that, let us briefly discuss an interesting hypothetical scenario of poling of host–guest systems at low temperatures. It takes into account the local inhomogeneity of the polymer matrix and the relation between characteristic spatial scales of local voids and of guest molecules. This topic was recently studied by one of us using Monte Carlo simulations^47^, leading to the conclusion that voids form scale-free clusters with power-law distribution of sizes and complex fractal structure, with fractal dimension dependent on the size of a cluster. This leads, in general, to a complex dynamics of guest molecules with sufficiently small spatial dimensions^48^, since some of them occupy voids and display the dynamics of an unconstrained molecule. The characteristic linear sizes of voids were roughly estimated as 6-9 nm for PMA matrix^47^, thus enabling the unconstrained poling scenario presented in this paper. The only difference between high- and low temperature scenarios for small guest molecules is that the dynamical evolution of local polymer environment of an octupole is present in the former and absent in the latter case.

Finally, let us address the challenging topic of possible experimental implementation of the poling in nano-scale. The main experimental difficulty is related to the spatial scale. The SHG signal $E(2\omega )\propto s \, d_\text {eff}$, where *s* denotes light-matter interaction pathlength and $d_\text {eff}$ is the nonlinear coefficient. In classical macroscopic EFISH experiments, macroscopic pathlengths are responsible for the generation of a detectable SHG signal. The extrapolation to the nanometer-sized sample volume of the proposed nano-octupoling cell with diameter 120 nm is not obvious but, nevertheless, is possible. Namely, EFISH has been measured for 100 nm polymer thin-film^49^, SHG signal from single nanocrystals^50,51^ and even from single molecules^52,53^ can be detected. For example, SHG from single nanocrystals of KTP (size 80 nm) was observed^51^. In this context it should be pointed out that the nonlinear coefficient $d_\text {eff}$ of KTP is of about 2-20 pm/V, at least 2 orders of magnitude higher than the value achievable in a system of non-interacting small octupolar molecules. Nevertheless, a very weak second harmonic signal (even single photons) can be detected using a relatively simple equipment^54^.

## Supplementary Information

Supplementary information.

## Data Availability

No datasets were generated or analysed during the current study.
