# Supplementary material for: Nanoscale ordering of planar octupolar molecules for nonlinear optics at higher temperatures
Source: Sci Rep. 2021 Jan 26;11:2234. doi: 10.1038/s41598-021-81676-9 (PMC7838211; doi:10.1038/s41598-021-81676-9)
Supplement: Supplementary file 1 — Supplementary information. [file 41598_2021_81676_MOESM1_ESM.pdf]

SUPPLEMENTARY INFORMATION

Nanoscale ordering of planar octupolar molecules for nonlinear optics at higher temperatures

Michał Jarema*,[1] Antoni C. Mituś,[2] and Joseph Zyss[3]
[1)] *Department of Semiconductor Materials Engineering, Wrocław University of Science and Technology, Wybrzeże Wyspiańskiego 27, 50–370 Wrocław, Poland*
[2)] *Department of Theoretical Physics, Wrocław University of Science and Technology, Wybrzeże Wyspiańskiego 27, 50–370 Wrocław, Poland*
[3)] *LUMIN Laboratory and Institut d'Alembert, Ecole Normale Supérieure Paris-Saclay, CNRS, Université Paris-Saclay, 4, avenue des Sciences, Gif-sur-Yvette, France*
**michal.jarema@pwr.edu.pl*

QUANTUM-CHEMICAL VALIDATION OF THE MODEL

The choice of point-charge model for octupolar molecules implies that interactions with an external electric field are of purely electrostatic nature. However, quantum effects can, potentially, introduce corrections. In this Section we use some simple methods of QChem to address this issue.

Octupole moment

Contributions to the octupole moment come from both electrons and nuclear charges. The electronic part was calculated using DFT in the *Dalton* program.[1,2] The electronic charge density, obtained from the solution of Kohn–Sham equations,[3] was numerically integrated to calculate the Cartesian components of the electronic contribution to the octupole moment, see Eq. (1) of the main text. The calculations used the DFT hybrid functional B3LYP and cc–pVDZ basis set. All calculations in this Appendix were done for a planar geometry of the TATB molecule optimized with the use of the B3LYP/6–31G(d) method. The magnitude of the total octupole moment (including electronic and nuclear contribution) was found to be

$$\|\mathcal{O}\| \approx 28.8\, e\text{Å}^3. \tag{1}$$

In the point-charge octupole model, the octupole moment is specified by two parameters, q and d (see Eq. (2) of the main text). In this paper we use $q = 0.66\ e$, $d = 2.44$ Å. Other combinations (e.g. $q = 0.5\ e$, $d = 2.67$ Å) have negligible impact on quantitative results. The parameters of a typical octupole used in Ref. 4 yield the value $||\mathcal{O}|| = 6\ e\text{Å}^3$. The calculated octupole moment of TATB is thus nearly five times larger, which increases the energy barrier accordingly (see Eq. (5) of the main text).

Energy barrier: quantum corrections

The energy barrier for the octupolar TATB molecule at the center of the poling cell (see Fig. 2 of the main text) was roughly estimated using QChem methods and compared to the barrier predicted in the point charges poling scheme. The QChem computations of energy of TATB molecule were done at the B3LYP/cc–pVDZ level, without optimalization of the molecular structure. The classic formula for the energy barrier of a model 6AO is given by Eq. (5) of the main text. We have found that both barriers have similar values even for much stronger poling fields than those used in this study, represented by four sets of values of Q and R in Table S1.

Q [e]	1	8	1/2	1
R [nm]	4	4	2	2
$\Delta E_{\text{QChem}}/k_{\text{B}}$ [K]	28.4	227	231	462
$\Delta E/k_{\text{B}}$ [K]	28.2	226	227	454

TABLE S1. Energy barriers computed by QChem methods ($\Delta E_{\text{QChem}}/k_{\text{B}}$) and $\Delta E/k_{\text{B}}$ predicted by the classic 6AO model.

GROUND STATE

Figure 1 shows the ground state orientation $\varphi_0(\vec{r})$ and the energy barrier $\Delta E(\vec{r})/k_B$ in the poling cell (k_B denotes Boltzmann constant). Those functions have been calculated analytically by direct evaluation of $E(\vec{r}, \varphi)$, in contrast to Ref. 4, where Monte Carlo simulations were used. The ground state orientation is inhomogeneous since the poling potential differs from the pure octupolar potential.[5] Nevertheless, in a wide central region, say $r < R/2$, the poling conditions are approximately homogeneous: $\varphi_0(\vec{r}) = 90° \pm 10°$. On leaving the center and approaching the cell boundary deviations from an homogeneous order become significant. The vortexes (*i.e.* points where the orientation is indefinite due to vanishing energy barrier) are located at distance $r \approx 0.58R$, similar to that in the electrode poling cell.[4]

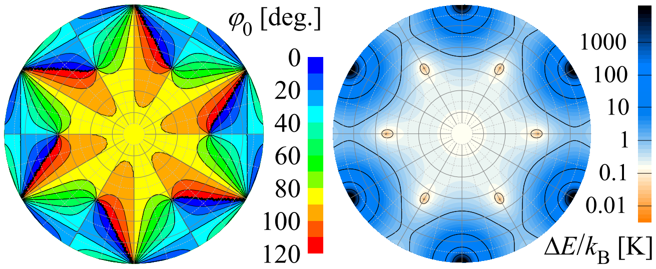

FIG. 1. Ground state orientation $\varphi_0(\vec{r})$ (left) and the energy barrier $\Delta E(\vec{r})/k_B$ (right) in the six point charges poling cell. The scale of the energy barrier was calculated for $Q = 300$ e and $R = 60$ nm.

[1] K. Aidas et al., WIREs Comput. Mol. Sci. **4**, 269 (2014).
[2] *Dalton*, a molecular electronic structure program, Release v2011 (2011), see http://daltonprogram.org.
[3] L. Piela, *Ideas of quantum chemistry*, Elsevier, Amsterdam, 2nd edition, 2014.
[4] A. C. Mituś, G. Pawlik, and J. Zyss, J. Chem. Phys. **135**, 024110 (2011).
[5] M. Jarema, A. C. Mituś, and J. Zyss, Acta Phys. Pol. B **43**, 1017 (2012).
